# A Six-Week Multimodal Functional Training Program Improves Physical Function and Modulates Redox Homeostasis in Older Women

**DOI:** 10.3390/medsci14050538

**Published:** 2026-09-01

**Authors:** Gábor Papp, Attila Csaba Arany, Ádám Diós, Ágnes Gyetvai, Andrea Nagy, Zoltán Csiki, László Balogh

**Affiliations:** 1 Institute of Sport Sciences, University of Debrecen, 4032 Debrecen, Hungary; aranyattila4@gmail.com (A.C.A.); balogh.laszlo@sport.unideb.hu (L.B.); 2 Division of Clinical Immunology, Institute of Internal Medicine, Faculty of Medicine, University of Debrecen, 4032 Debrecen, Hungary; dios.adam@med.unideb.hu (Á.D.); gyetvaia@med.unideb.hu (Á.G.); nagy.andrea1@med.unideb.hu (A.N.); csiki@med.unideb.hu (Z.C.); 3 Doctoral School of Medical Sciences, University of Debrecen, 4032 Debrecen, Hungary

**Keywords:** ageing, exercise, oxidative stress, antioxidant enzymes

## Abstract

**Background/Objectives**: Age-related increases in oxidative stress and chronic low-grade inflammation contribute to skeletal muscle deterioration, impaired physical performance, and loss of functional independence in older adults. Regular exercise may counteract these processes by improving both musculoskeletal function and systemic redox homeostasis; however, the effects of multimodal functional exercise on antioxidant defence mechanisms and innate immune redox regulation remain incompletely understood. Therefore, the aim of the present study was to investigate changes associated with a six-week multimodal functional exercise program in body composition, muscle strength, physical performance, antioxidant defence and phagocyte oxidative burst activity in older women. **Methods**: Seventeen physically inactive women aged over 65 years participated in a six-week functional training program consisting of aerobic, resistance, balance, coordination, and proprioceptive exercises performed twice weekly. Body composition, handgrip strength, and Short Physical Performance Battery (SPPB) scores were assessed before and after the intervention. In addition, serum antioxidant enzyme activities, total antioxidant capacity, lipid peroxidation markers, nitrite/nitrate concentrations, and phagocyte oxidative burst activity were determined. **Results**: Following the intervention, improvements were observed in skeletal muscle mass, muscle percentage, handgrip strength, and SPPB scores, while body fat mass and body fat percentage decreased. Catalase activity increased, whereas superoxide dismutase activity, glutathione peroxidase activity, and lipid peroxidation markers remained unchanged. Serum nitrite/nitrate concentrations increased following the intervention. Although neutrophil counts did not change, phagocyte oxidative burst activity decreased significantly. **Conclusions**: These findings indicate that a six-week multimodal functional exercise program was associated with favourable changes in body composition, enhanced muscle strength, and improved functional performance in previously inactive older women. In parallel, favourable changes in antioxidant defence, nitric oxide metabolism, and immune-related redox regulation were also observed following the intervention, supporting the potential role of multimodal functional training as an effective non-pharmacological strategy for promoting healthy ageing.

## 1. Introduction

Population ageing has become a major demographic and public health challenge in developed countries over recent decades. Although life expectancy has improved considerably due to advances in healthcare and living conditions, ageing is accompanied by a progressive decline in physiological reserve and functional capacity, resulting in reduced independence, increased healthcare utilization, and impaired quality of life. Ageing is associated with complex biological alterations affecting multiple organ systems. Among these changes, increased oxidative stress and chronic low-grade inflammation play central roles in the development of age-related functional decline [1]. The imbalance between reactive oxygen species (ROS) production and antioxidant defence mechanisms contributes to cellular damage, impaired tissue regeneration, and the progression of several chronic diseases [2]. In skeletal muscle, these processes are closely linked to the age-related progressive loss of muscle mass, strength, and physical performance [3]. These mechanisms contribute to the development of sarcopenia, a condition strongly associated with an increased risk of falls, fractures, disability, hospitalization, and mortality, representing a major challenge for healthy ageing [4]. Age-related declines in muscle mass, strength, and physical performance are driven by multiple factors, including physical inactivity, chronic inflammation, oxidative stress, endocrine alterations, impaired muscle regeneration and neuromuscular degeneration [5,6]. Ageing is accompanied by a progressive decline in immune function, which promotes the development of a chronic low-grade inflammatory state known as inflammageing [7], which contributes to mitochondrial dysfunction, leading to reduced ATP production and excessive generation of reactive oxygen species (ROS) [8]. The resulting oxidative stress further amplifies mitochondrial damage and activates proteolytic pathways, including the ubiquitin–proteasome system, thereby accelerating skeletal muscle atrophy and functional decline [9]. Age-related increases in pro-inflammatory state and excessive ROS production contribute to muscle protein degradation, reduced protein synthesis, and impaired satellite cell function, ultimately leading to progressive deterioration of muscle structure and function [10,11]. However, current concepts emphasize that healthy ageing depends not merely on limiting ROS production but on preserving redox homeostasis. Physiological levels of ROS function as signalling molecules regulating mitochondrial biogenesis, antioxidant enzyme expression and cellular adaptation. Ageing is fundamentally associated with a progressive loss of this adaptive redox flexibility, leading to increased vulnerability to oxidative damage [12]. Consequently, interventions capable of improving muscle function while simultaneously modulating redox adaptation may have particular relevance in older populations.

Regular physical activity is considered one of the most effective non-pharmacological strategies for preserving functional capacity and promoting healthy ageing [13]. Exercise has been shown to improve cardiovascular and metabolic health, enhance muscle strength and physical performance, reduce the risk of chronic diseases, and stimulate endogenous antioxidant defence systems [14]. While acute exercise may transiently increase ROS production, regular exercise training induces adaptive responses that improve antioxidant capacity and resistance to oxidative stress [15,16]. Notably, exercise-induced ROS generation is increasingly recognized as a physiological signal rather than solely a harmful by-product, since it activates adaptive cellular responses, including nuclear factor erythroid 2-related factor 2 (Nrf2)-mediated antioxidant defence mechanisms [17]. Previous studies suggest that multimodal exercise programs combining aerobic, resistance, balance, and flexibility training may be particularly effective in improving muscle function and physical performance in older adults and individuals with sarcopenia [18,19]. Functional training has emerged as a comprehensive exercise approach that integrates strength, balance, coordination, proprioception, and cardiorespiratory fitness through movements resembling activities of daily living [20]. Such programs may be especially beneficial for older adults because they simultaneously target multiple components of physical function that are essential for maintaining independence and reducing fall risk. Nevertheless, although the effects of exercise on physical performance have been extensively investigated, less is known about the influence of multimodal functional exercise programs on oxidative stress markers in older adults. In addition to its well-established effects on skeletal muscle and vascular function, ageing is also associated with profound alterations in innate immunity. Age-related dysregulation of neutrophil and phagocyte function contributes to chronic low-grade inflammation (inflammageing) and enhanced production of reactive oxygen species, both of which may promote tissue injury and functional decline [21,22]. Although the beneficial effects of exercise on physical performance are well documented, considerably less is known about whether multimodal functional exercise training can modulate phagocyte-derived ROS production and redox regulation in older adults.

Therefore, the aim of the present study was to investigate the effects of a six-week functional exercise program on body composition, muscle strength, physical performance, oxidative stress-related biomarkers, and phagocyte oxidative burst activity in physically inactive older women with reduced physical performance.

## 2. Materials and Methods

### 2.1. Participants

Seventeen community-dwelling older women with reduced physical performance were enrolled in the present study (mean age: 71.24 ± 4.55 years; mean BMI: 28.81 ± 6.05 kg/m^2^). Inclusion criteria comprised age ≥ 65 years, an SPPB score ≤ 10 points, and the absence of participation in structured physical exercise during the six months preceding enrolment. Participants were recruited through a local retirement association in Debrecen, where the six-week exercise programme was advertised. The number of volunteers substantially exceeded the required sample size, and eligible participants were selected according to the predefined inclusion and exclusion criteria. This recruitment strategy therefore resulted in a convenience sample of community-dwelling older women. All participants were non-smokers, and had not followed any special diet or used vitamin supplements for at least three months before enrolment. Moreover, exclusion criteria included ongoing viral or bacterial infection, allergic or autoimmune diseases, malignancy, alcohol or drug abuse, psychiatric disorders, poor compliance and any changes in dietary habits or use of dietary supplements during the study period. Eligibility and compliance were assessed through a structured medical interview conducted by a physician at baseline and at the end of the study.

### 2.2. Exercise Protocol

All participants attended 90 min functional exercise sessions twice a week, in the morning (sessions started between 8 and 9 a.m.) for six weeks under stable climatic conditions (20–22 °C) at UniFit Fitness and Gym Centre in Debrecen. The exercise protocol was designed according to the American College of Sports Medicine (ACSM) position stand on “Exercise and physical activity for older adults” [23]. To ensure precise and safe execution of the exercises, all training sessions were supervised by a physiotherapist. Each training session began with 10 min of warm-up and mobility exercises. This was followed by 20 min balance and proprioceptive training phase, which included various gait tasks (e.g., tandem, slalom, backward, toe-, heel-, and lateral-foot walking) performed on progressively challenging support surfaces, including narrow and unstable foam surfaces, as well as tasks requiring rapid adaptation to unexpected verbal cues and changes in movement patterns. Proprioceptive control was further enhanced through weight-shifting exercises and other balance tasks. Subsequently, participants performed 20 min of aerobic exercises on a treadmill (Star Trac Model 8TRx, Core Health & Fitness LLC, Vancouver, NE, USA), elliptical trainer (Star Trac Model 8CT, Core Health & Fitness LLC) or stationary bicycle (Activate Series Recumbent Lifecycle^®^ Exercise Bike, Life Fitness-Brunswick Corporation, Lake Forest, IL, USA). Maximum heart rate (HRmax) was estimated using the Tanaka equation (HRmax = 208 − 0.7 × age) [24] and exercise intensity was prescribed as a percentage of the estimated HRmax. In the first 10 min, low intensity aerobic exercises were carried out and the intensity was enhanced up to 40–50% of HRmax in order to protect the joints; thereafter, the intensity was increased up to 50–60% of HRmax for the next 10 min. Heart rate was monitored by the heart rate measurement system of the device. In the next 20 min block, cyclic exercises were used to prepare the large muscles for muscle force development. Muscle groups of the upper, lower limbs and core muscles were trained using TRX suspension exercises, including TRX Squat, TRX Low Rows, TRX Push-Up, and TRX Standing Hip Drop exercises. Exercise intensity was individualized by adjusting the inclination angle between 10° and 45°, enabling participants to complete 12 intensive repetitions. To ensure adequate rest periods between repetitions, participants performed the exercises in pairs. To further strengthen the core musculature, participants completed an additional 10 min of Fitball exercises following the TRX block. Fitball exercises were performed with assistance during the first three weeks and independently during the final three weeks. All training was finished with a 10 min stretching section. Table 1 summarizes the detailed training protocol. Attendance at all scheduled training sessions was expected throughout the intervention. All participants completed all 12 supervised training sessions (100% adherence), and no participants withdrew or were excluded because of illness, infection, injury, or other health-related reasons during the intervention period.

### 2.3. Body Composition Analyses

Body composition was assessed using an InBody 270 device (InBody, Seoul, Republic of Korea) following the manufacturer’s recommended measurement protocol. To ensure standardized measurement conditions, assessments were conducted after an overnight fast, between 8:00 and 9:00 a.m., and participants were instructed to empty their bladder immediately before testing in order to minimize the influence of hydration status and diurnal variation on body composition parameters. After the manual input of basic data, including height, sex, and age, the measurements of body composition took approximately 30 s per participant. Besides the body weight, the analysis provides a comprehensive assessment of the mass of skeletal muscles and body fat regarding the whole body as well as its different parts including upper and lower extremities and trunk. Body Mass Index (BMI) and Skeletal Muscle Mass Index (SMI) were also calculated for each measurement. SMI reflects appendicular skeletal muscle mass normalized to height squared (kg/m^2^).

### 2.4. Assessment of Physical Performance

Short Physical Performance Battery (SPPB) test was used for the assessment of the severity of decline in physical performance. SPPB evaluation includes a group of measures that combines the results of the gait speed, chair stand and balance tests. Each component was scored from 0 to 4, resulting in a total SPPB score ranging from 0 to 12, with lower scores indicating poorer physical performance. Different components of the SPPB evaluate distinct aspects of physical function. Walking speed primarily reflects mobility, static balance assesses postural control, whereas repeated chair rises provide an estimate of lower-extremity muscle strength, coordination, balance, and functional mobility [25].

### 2.5. Assessment of Handgrip Strength

Handgrip strength was measured using a CAMRY Digital Hand Dynamometer (Camry Scale, South El Monte, CA, USA). Participants were instructed to squeeze the device with maximal force, first using the right hand and then the left hand. Results were recorded in kilograms.

### 2.6. Blood Sampling and Analysis of Blood Cell Counts

Peripheral blood samples for laboratory analyses were collected from all participants at baseline and 72 h after the final exercise session to minimize the influence of transient acute exercise-induced oxidative stress responses. This timing was selected based on previous evidence showing that most circulating oxidative stress biomarkers return toward baseline within 24–72 h after exercise, thereby allowing a more reliable assessment of training-related physiological adaptations [26,27,28]. To ensure standardized sampling conditions, blood was drawn between 8:00 and 9:00 a.m. after participants had fasted overnight, thereby minimizing potential diurnal variation and the influence of recent food intake on the investigated markers. Venous blood samples were collected from a forearm vein into VACUETTE^®^ CAT Serum Separator Tubes, VACUETTE^®^ EDTA Tubes, and VACUETTE^®^ NH Sodium Heparin Tubes (Greiner Bio-One, Kremsmünster, Austria). All serum and plasma samples were visually inspected for haemolysis prior to biochemical analyses, and no visibly haemolysed samples were identified. Hematological parameters, including total neutrophil granulocyte and monocyte counts, were determined from EDTA-anticoagulated blood using a Sysmex XN-2000 Hematology Analyzer (Sysmex Europe GmbH, Norderstedt, Germany). Phagocyte oxidative burst activity was assessed from fresh heparin-anticoagulated whole blood. Serum and plasma samples were separated by centrifugation at 1000× *g* for 10 min, aliquoted, and stored at −80 °C until biochemical analyses.

### 2.7. Determination of Oxidative Stress Markers

Serum samples were used to evaluate catalase (CAT) and superoxide dismutase (SOD) activities, total antioxidant capacity (TAC), thiobarbituric acid reactive substances (TBARS), and nitrite/nitrate levels, whereas glutathione peroxidase (GPx) activity was analysed from plasma samples in accordance with the manufacturer’s validated assay protocol, which specifies plasma as the recommended sample matrix. CAT activity was determined using the Catalase Assay Kit (Cat. No. 707002), which quantifies formaldehyde generated during hydrogen peroxide degradation through a colorimetric reaction involving purpald. SOD activity was measured with the Superoxide Dismutase Assay Kit (Cat. No. 706002), based on the enzyme’s ability to eliminate superoxide radicals produced by xanthine oxidase. GPx activity was analysed using the Glutathione Peroxidase Assay Kit (Cat. No. 703102), where enzymatic activity is indirectly estimated through monitoring NADPH consumption in a glutathione reductase-mediated reaction. Total nitrite/nitrate concentrations were quantified using the Nitrate/Nitrite Colorimetric Assay Kit (Cat. No. 780001). In this procedure, nitrate present in serum is first enzymatically reduced to nitrite, followed by reaction with Griess reagents to produce a chromophoric compound detectable by spectrophotometry. Overall antioxidant capacity was evaluated with the Antioxidant Assay Kit (Cat. No. 709001), which assesses both enzymatic and non-enzymatic antioxidant components and expresses results as Trolox equivalents. Lipid peroxidation was measured using the TBARS (TCA Method) Assay Kit (Cat. No. 700870), based on the generation of a colored adduct when malondialdehyde (MDA) reacts with thiobarbituric acid under acidic conditions. All assay kits were obtained from Cayman Chemicals (Ann Arbor, MI, USA), and analyses were performed in accordance with the manufacturer’s protocols using a Labsystems Multiskan MS Type 352 microplate reader (Thermo Fisher Scientific, Waltham, MA, USA).

### 2.8. Assessment of Phagocyte Oxidative Burst Activity

The oxidative burst response of phagocytes was evaluated using a Berthold Autolumat Plus BL 953 luminometer (Berthold, Bad Wildbad, Germany) according to a previously published protocol [29]. Zymosan particles (Merck KGaA, Darmstadt, Germany) served as stimulants to induce oxidative burst activity, phosphate-buffered saline (Merck KGaA, Darmstadt, Germany) was used as a negative control, while phorbol 12-myristate 13-acetate (PMA; Merck KGaA, Darmstadt, Germany) functioned as a positive control. Luminol oxidation was monitored for 60 min at 37 °C by recording emitted photons within the 360–630 nm wavelength range at 5 min intervals. Chemiluminescence outcomes were expressed as Relative Light Units (RLU). Oxidative burst activity was evaluated by calculating the area under the curve (AUC) of the kinetic response curves normalized to 1000 neutrophil cells per sample. Although the whole-blood assay also reflects the contribution of monocytes, neutrophils constitute the predominant circulating phagocyte population and are generally regarded as the principal contributors to luminol-enhanced chemiluminescence in whole blood. Therefore, normalization to neutrophil count was considered an appropriate approach for standardizing oxidative burst responses between samples.

### 2.9. Statistical Analysis

Data analysis was performed using GraphPad Prism version 7.0 (GraphPad Software, San Diego, CA, USA). Descriptive statistics were illustrated as boxplots displaying the interquartile range (IQR), with medians indicated by the central line. The Shapiro–Wilk test was applied to determine data normality. Comparisons of pre- and post-exercise measurements were analyzed using paired *t*-tests under normal distribution assumptions, while the Wilcoxon signed-rank test was used for non-parametric data. Statistical significance was defined at *p* < 0.05. Because the study evaluated multiple outcomes across different physiological domains, no formal adjustment for multiple comparisons was applied. Consequently, the reported *p*-values should be interpreted with appropriate caution. Effect sizes and 95% confidence intervals are presented to facilitate interpretation of the magnitude and precision of the observed changes.

## 3. Results

### 3.1. Changes in Body Composition and Physical Performance

**Figure 1:**
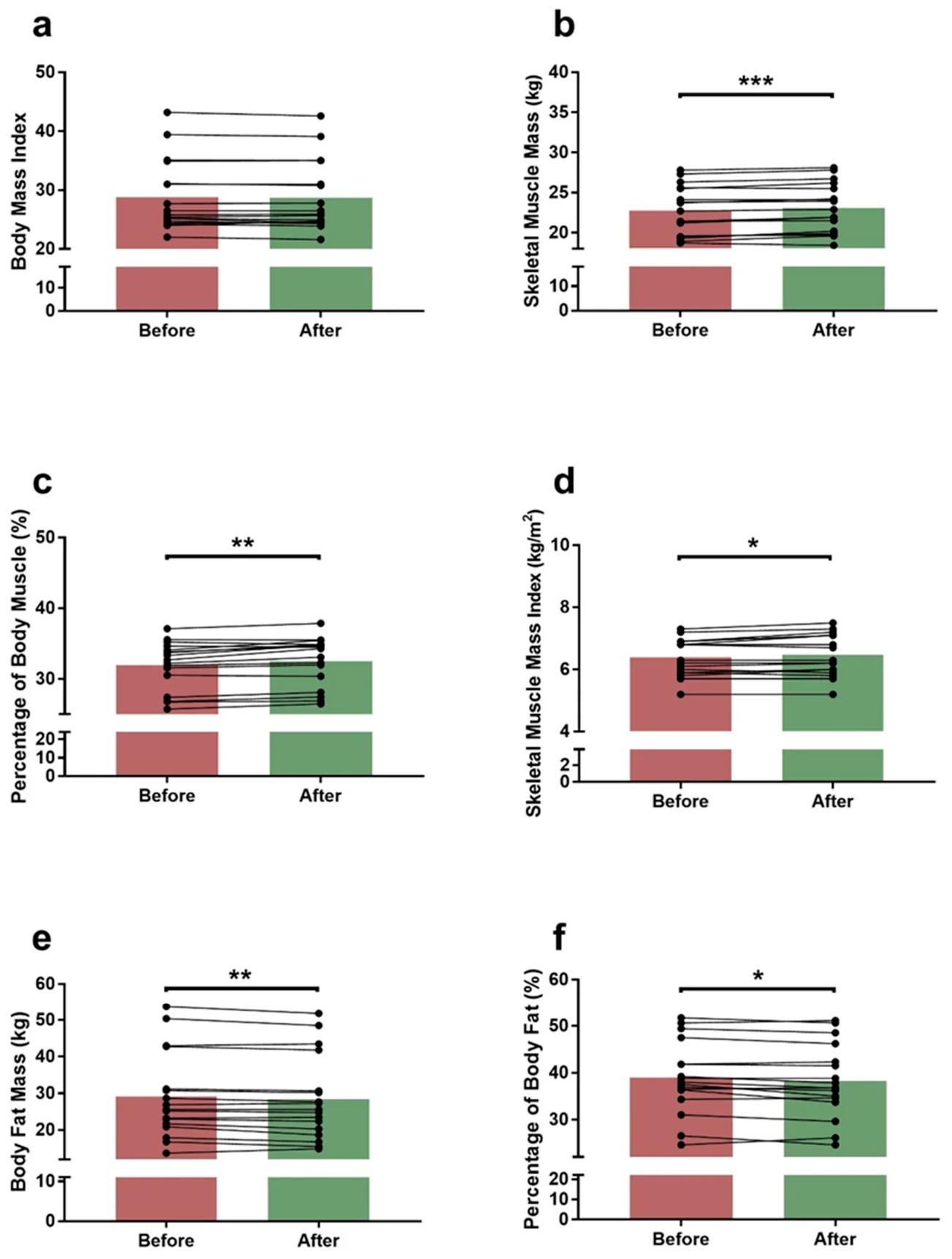
Body composition of the participants (*n* = 17) before and after the exercise intervention. (**a**) body mass index (BMI), (**b**) skeletal muscle mass, (**c**) percentage of body muscle, (**d**) skeletal muscle mass index, (**e**) body fat mass, (**f**) percentage of body fat values are represented as dots, while columns display mean. Normally distributed values (**b**–**f**) were analyzed by paired *t*-test, while non-normally distributed values (**a**,**c**) by Wilcoxon test. Statistically significant differences are indicated by * *p* < 0.05; ** *p* < 0.01; *** *p* < 0.001.

In order to assess the effects of regular exercise on the fitness level of participants, measurements on body composition and physical performance were performed at baseline and repeated 3 days after the last training session. BMI remained unchanged during the intervention; however, significant improvements were observed in body composition parameters. Skeletal muscle mass increased from 22.75 ± 3.04 kg to 23.09 ± 3.05 kg (*p* = 0.0006) percentage of body muscle increased from 31.93 ± 3.42% to 32.54 ± 3.47% (*p* = 0.0011), and SMI increased from 6.39 ± 0.60 kg/m^2^ to 6.48 ± 0.67 kg/m^2^ (*p* = 0.0216). In parallel, body fat mass decreased from 29.15 ± 11.69 kg to 28.47 ± 11.44 kg (*p* = 0.0092), and percentage of body fat decreased from 38.96 ± 7.76% to 38.24 ± 7.81% (*p* = 0.0181) by the end of the exercise program (Figure 1).

**Figure 2:**
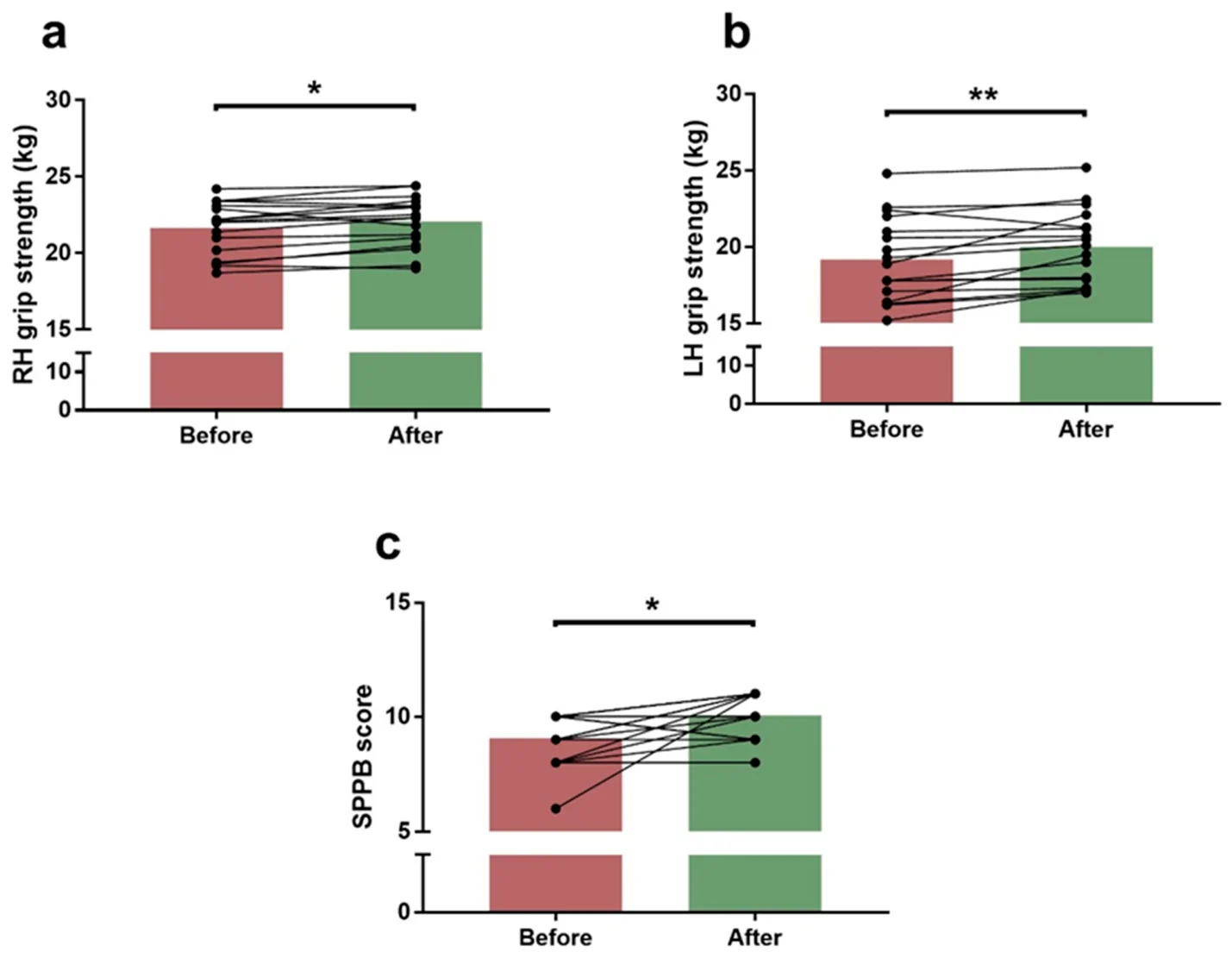
Physical performance of the participants (*n* = 17) before and after the exercise intervention. (**a**) Right hand (RH) grip strength, (**b**) Left hand (LH) grip strength, (**c**) Short Physical Performance Battery (SPPB) values are represented as dots, while columns display mean. Normally distributed values (**a**,**b**) were analyzed by paired *t*-test, while non-normally distributed values (**c**) by Wilcoxon test. Statistically significant differences are indicated by * *p* < 0.05; ** *p* < 0.01.

In addition, positive changes were observed in muscle strength, as improvements were observed in both right- and left-hand grip strength. Participants showed significant increases in right-hand grip strength from 21.65 ± 1.73 kg to 22.06 ± 1.65 kg (*p* = 0.0131) and left-hand grip strength from 19.18 ± 2.72 kg to 20.01 ± 2.43 kg (*p* = 0.0063) compared with baseline values (Figure 2a,b). Physical performance improved as well, as indicated by higher SPPB scores after the intervention (9.06 ± 1.14 vs. 10.06 ± 0.97, respectively; *p* = 0.0142) (Figure 2c). Detailed descriptive statistics are presented in Supplementary Table S1.

### 3.2. Changes in Enzymatic Antioxidant Activities and Levels of Oxidative Stress Markers

**Figure 3:**
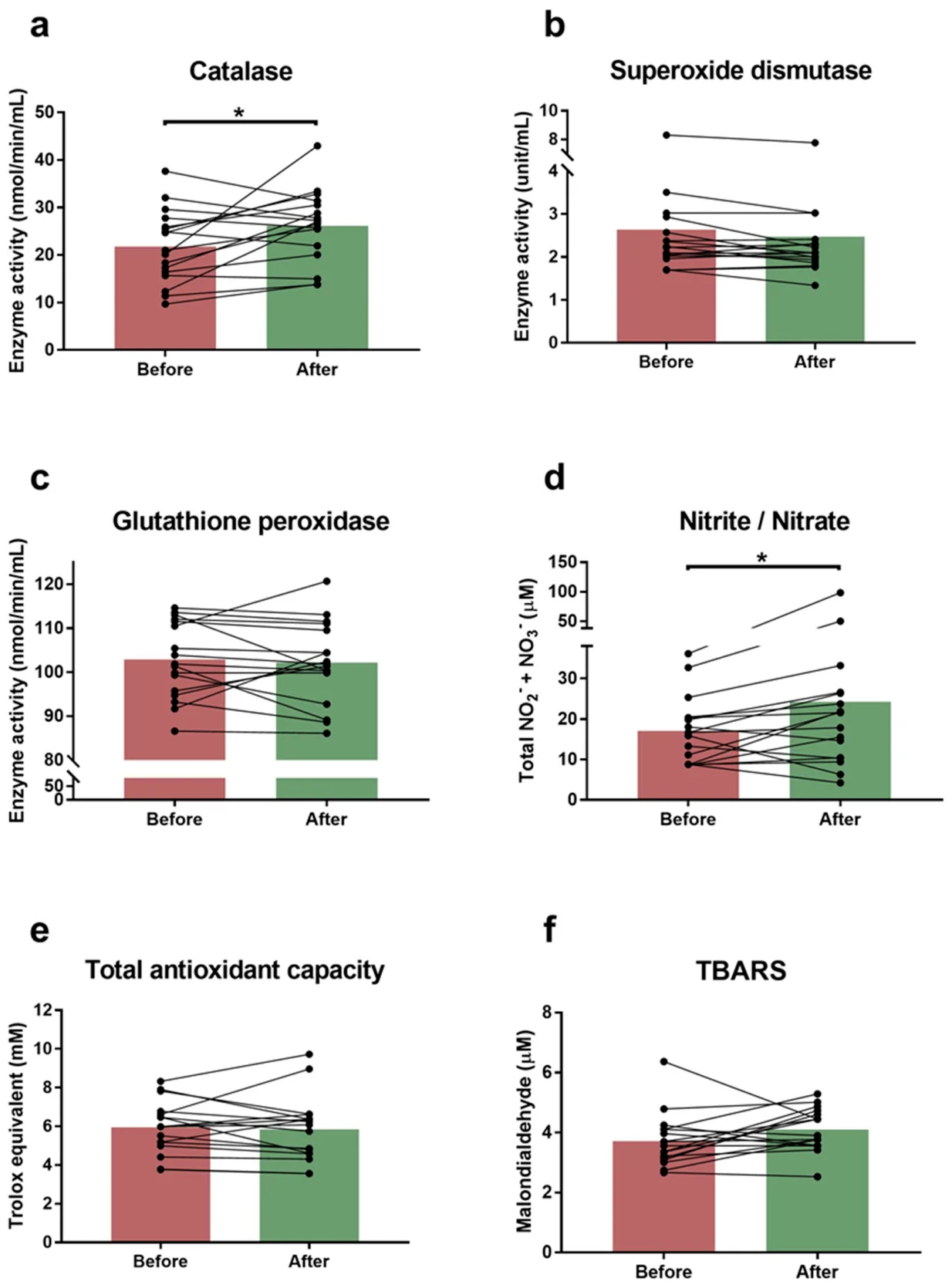
Antioxidant and oxidative stress-related markers in the peripheral blood of the participants (*n* = 17) before and after the exercise intervention. Panels show (**a**) catalase (CAT) activity, (**b**) superoxide dismutase (SOD) activity, (**c**) glutathione peroxidase (GPx) activity, (**d**) total nitrite/nitrate concentration, (**e**) total antioxidant capacity (TAC), and (**f**) thiobarbituric acid reactive substances (TBARS) levels. Individual values are represented as dots, while columns display mean. Normally distributed values (**a**,**c**,**e**) were analyzed by paired T test, while non-normally distributed values (**b**,**d**,**f**) by Wilcoxon test. Statistically significant differences are indicated by * *p* < 0.05.

Catalase activity increased significantly after the exercise intervention (21.78 ± 7.70 nmol/min/mL vs. 26.15 ± 7.60 nmol/min/mL, respectively; *p* = 0.0289) (Figure 3a), whereas SOD and GPx activities did not change significantly (Figure 3b,c). The total nitrite/nitrate concentrations showed significant elevation at the end of the exercise program (17.03 ± 8.33 µM vs. 24.22 ± 22.1 µM, respectively; *p* = 0.0348) (Figure 3d). Total antioxidant capacity and TBARS level did not change significantly (Figure 3e,f).

### 3.3. Oxidative Burst Activity of Phagocytes

**Figure 4:**
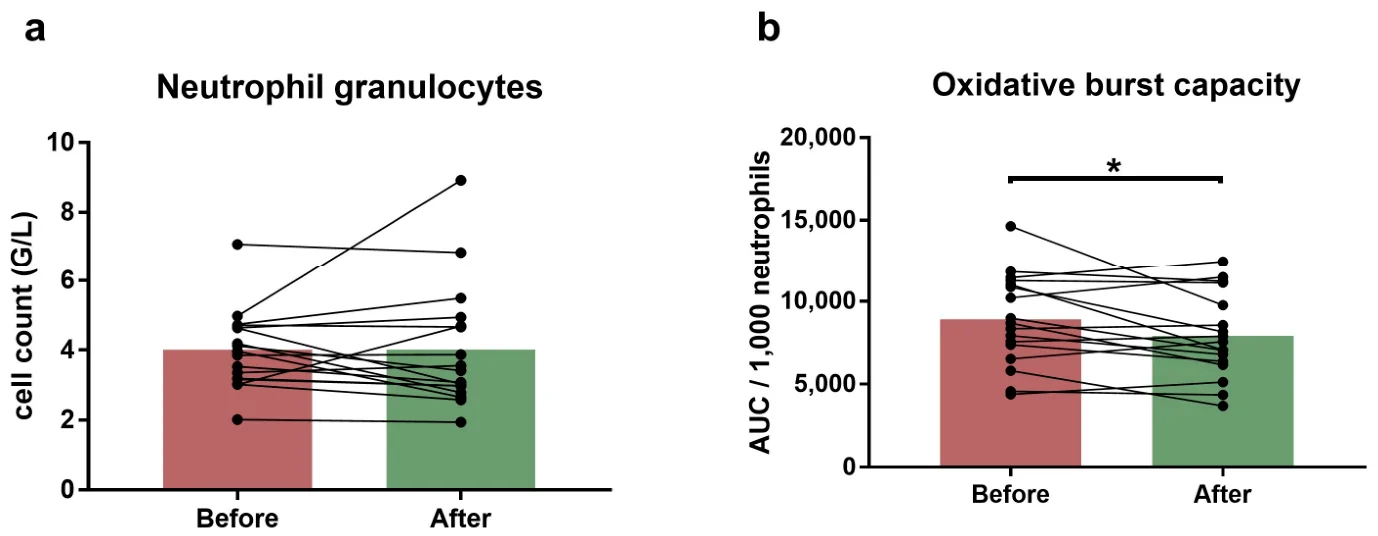
Changes in (**a**) peripheral blood neutrophil counts and (**b**) stimulated oxidative burst capacity in study participants (*n* = 17) before and after six-week exercise intervention. Individual values are represented as dots, while columns display mean. Normally distributed values (**b**) were analyzed by paired *t*-test, while non-normally distributed values (**a**) by Wilcoxon test. Statistically significant differences are indicated by * *p* < 0.05.

No significant changes were observed in the absolute counts or percentages of neutrophils and other leukocyte subsets after the six-week intervention. Likewise, the absolute monocyte count remained unchanged following the intervention (before: 0.53 ± 0.13 G/L vs. after: 0.52 ± 0.14 G/L, respectively; *p* = 0.735). Absolute neutrophil counts are presented in Figure 4a. In contrast, stimulated oxidative burst capacity, expressed as AUC normalized to 1000 neutrophils, decreased significantly by the end of the exercise program (8903 ± 2777 vs. 7933 ± 2564, respectively; *p* = 0.0418) (Figure 4b).

## 4. Discussion

Healthy ageing is strongly dependent on the preservation of functional capacity in later life. Therefore, in an increasingly ageing society, the identification of effective interventions capable of counteracting age-related functional decline has become a major public health priority. The deterioration of functional status is largely attributable to age-related alterations in skeletal muscle, antioxidant defence mechanisms, and other physiological systems. Mitochondrial dysfunction, increased oxidative stress, impaired antioxidant responses, chronic inflammation, and altered myokine signalling have all been implicated in the structural and functional deterioration of skeletal muscle associated with ageing. Regular participation in physical activity is essential for preserving functional capacity and supporting healthy ageing. Higher levels of physical activity have been associated with a reduced rate of age-related loss of muscle mass and strength, thereby contributing to the preservation of physical function in older individuals [30]. In our present study, in addition to assessing changes in physical condition following a six-week moderate-intensity functional exercise program, we also evaluated changes in antioxidant defence mechanisms in older women.

By the end of the intervention, significant improvements were observed in several physical parameters. Favourable alterations in body composition were detected, including a reduction in body fat mass and an increase in skeletal muscle mass. These changes were accompanied by improvements in muscle strength, reflecting enhanced physical performance in the study population. Our findings are consistent with previous observations demonstrating the beneficial effects of regular exercise on the improvement of muscle mass and strength [19,20]. The increase in SPPB score was consistent with previous reports suggesting that integrated exercise interventions can effectively improve physical performance and reduce the risk of falls in older adults [31]. Beyond statistical significance, the clinical relevance of these findings should also be considered. The mean SPPB score increased by 1.0 point following the intervention, reaching the threshold proposed for a substantial clinically meaningful improvement in older adults [32], suggesting a functionally relevant enhancement in lower-extremity physical performance. Although handgrip strength also increased significantly, the magnitude of change remained below the currently suggested range (approximately 5.0–6.5 kg) for a clinically meaningful difference [33]. The clinical relevance of the observed body-composition changes is less clear, as validated thresholds for clinically meaningful short-term changes in InBody-derived body-composition parameters have not been established in a comparable population. Nevertheless, the consistent improvements across skeletal muscle and adiposity measures may represent early favourable adaptations following the relatively short intervention. Longer controlled studies are needed to determine whether these changes increase further and translate into sustained clinically meaningful benefits. Taken together, these findings suggest that even a relatively short-term functional training program may lead to clinically relevant improvements in lower-extremity physical performance while also promoting favourable early adaptations in muscle strength and body composition in older women.

Following appropriate physiological adaptation, regular exercise enhances antioxidant defence mechanisms, attenuates pro-inflammatory processes, and promotes anabolic signalling pathways and mitochondrial biogenesis within skeletal muscle. Moreover, exercise exerts systemic anti-inflammatory effects by reducing circulating inflammatory cytokines and oxidative stress markers [34]. In the vascular system, physical activity improves endothelial function and reduces arterial stiffness through the attenuation of oxidative and inflammatory signalling pathways, accompanied by enhanced antioxidant enzyme activity and increased nitric oxide (NO) bioavailability [35]. Ageing is characterized by a gradual decline in antioxidant defence capacity, accompanied by increased oxidative stress and chronic low-grade inflammation, which further promote the accumulation of reactive oxygen species. The enzymatic antioxidant defence system plays a critical role in counteracting the harmful effects of reactive oxygen species; among the most important antioxidant enzymes are SOD CAT and GPx. In the present study, the observed increase in CAT activity may reflect an adaptive hormetic response to regular exercise. Moderate exercise-induced ROS generation is increasingly recognized as a physiological signalling mechanism (‘oxidative eustress’) that activates redox-sensitive transcription factors such as Nrf2 and nuclear factor kappa light chain enhancer of activated B cells (NF-κB), leading to enhanced endogenous antioxidant defence [17]. The selective increase in CAT activity may indicate that CAT represents one of the earliest adaptive antioxidant responses to exercise training. Similar findings have been reported following short-term HIIT interventions, where catalase activity increased despite the absence of significant changes in other antioxidant enzymes [36,37]. The absence of changes in TBARS suggests that exercise-induced oxidative stress did not exceed the threshold required to induce measurable lipid peroxidation. Although TBARS remains one of the most widely used assays for assessing lipid peroxidation in exercise physiology, it is less analytically specific than chromatographic methods or F2-isoprostane measurements. Therefore, these findings should be interpreted with consideration of the analytical characteristics of the assay. Following the intervention, serum nitrite/nitrate concentrations increased significantly. Endothelial dysfunction and arterial stiffness are among the most common age-related vascular alterations [38]. Exercise is known to stimulate NO production and release, thereby contributing to vascular homeostasis through its vasodilatory and anti-atherogenic properties. Increased NO bioavailability improves tissue perfusion and enhances oxygen and nutrient delivery to peripheral tissues and organ [39]. Beyond its vasodilatory effects, increased NO bioavailability may contribute to improved skeletal muscle perfusion, oxygen delivery, mitochondrial function, and exercise adaptation [40]. The concomitant increase in CAT activity and nitrite/nitrate concentrations may indicate a shift in redox-related and nitric oxide metabolic pathways following the intervention.

Another important observation of the present study was the significant reduction in stimulated phagocyte oxidative burst activity despite unchanged neutrophil and leukocyte counts. Ageing is characterized by chronic low-grade inflammation and redox imbalance, and previous studies have suggested that spontaneous ROS production by neutrophils may increase with age, representing an aspect of age-associated immune dysregulation [21]. Phagocyte-derived ROS are essential for antimicrobial host defence; however, excessive or dysregulated ROS generation may also contribute to systemic oxidative stress and tissue damage. In this context, the combined findings of increased catalase activity, elevated nitrite/nitrate concentrations, and reduced stimulated oxidative burst may reflect alterations in immune-redox regulation following the intervention. At present, no clinically established threshold or minimal clinically important difference has been defined for luminol-enhanced phagocyte oxidative burst activity. Therefore, the observed reduction may reflect altered phagocyte redox responsiveness; however, its physiological significance remains uncertain because no direct assessment of inflammatory mediators, immune competence, or susceptibility to infection was performed. Nevertheless, because reactive oxygen species generated during the oxidative burst play an essential role in antimicrobial defence, an excessive reduction in oxidative burst activity could theoretically compromise host defence. In the present study, however, leukocyte, monocyte and neutrophil counts remained unchanged, and the observed reduction was relatively modest. Previous studies in older adults suggest that regular physical activity may preserve or improve neutrophil phagocytic activity [41]. In particular, long-term resistance training in older women was associated with higher neutrophil phagocytic activity without altering oxidative burst activity, although differences in assay methodology should be considered when comparing these findings with the present results [42]. Taken together, these observations and our present findings suggest that the reduction in stimulated oxidative burst should be interpreted cautiously as reflecting altered phagocyte redox responsiveness rather than evidence of generalized immunosuppression. Nevertheless, the physiological and clinical significance of this finding remains uncertain and should be clarified in future studies incorporating a broader assessment of immune function.

The present findings should also be interpreted in light of some limitations. The single-group pre-post design, the relatively small sample size, the inclusion of only community-dwelling older women, the relatively short intervention period warrant cautious interpretation of the findings and confirmation in longer-term and larger controlled studies. Additionally, although several biomarkers of redox homeostasis were assessed, the biomarker panel was limited and did not include inflammatory cytokines or more comprehensive measures of immune function. Consequently, the physiological significance of the observed changes in phagocyte oxidative burst requires further investigation.

In summary, our findings indicate that a relatively short, six-week multimodal functional training program was associated with significant improvements in body composition, muscle strength, and physical performance in previously inactive older women. Beyond these functional benefits, the intervention was accompanied by favourable adaptations in redox-related parameters, including increased CAT activity and nitrite/nitrate concentrations, together with a reduction in stimulated phagocyte oxidative burst activity. Collectively, these findings suggest that regular functional exercise may contribute not only to the preservation of physical function but also to the modulation of antioxidant defence mechanisms and immune-related redox regulation in older adults. Given the established role of oxidative stress and impaired redox regulation in age-related tissue damage, including skeletal muscle deterioration, these findings are consistent with the concept that regular multimodal exercise may contribute to healthy ageing not only through functional adaptations but also by promoting a more favourable systemic redox environment. 

## 5. Conclusions

Our study indicates an improvement in body composition, muscle strength, and physical performance in previously inactive older women after a six-week functional exercise program. In parallel, the changes observed in antioxidant defence, nitric oxide metabolism, and phagocyte redox responsiveness suggest that the benefits of functional training extend beyond improvements in musculoskeletal performance and are accompanied by adaptations in systemic redox regulation.

## Figures and Tables

**Table 1:** Detailed training protocol.

Duration	Exercise Modality	Intensity Level 1 (Weeks 1–3)	Intensity Level 2 (Weeks 4–6)
10 min	warm-up and mobility exercises	low complexity	increased complexity
20 min	coordination, balance and proprioceptive exercises	basic balance tasks	advanced balance tasks
20 min	aerobic exercises with treadmill/elliptical trainer/bicycle	40–50% HRmax (10 min) 50–55% HRmax (10 min)	40–50% HRmax (10 min) 55–60% HRmax (10 min)
20 min	TRX Squat	3 sets of 12–15 reps	-
	TRX Single Leg Squat	-	3 sets of 10 reps per leg
	TRX Low Rows	3 sets of 12–15 reps (range 10–45°)	-
	TRX Single Arm Low Rows	-	3 sets of 10 reps per arm (range 10–45°)
	TRX Push-Up	3 sets of 12–15 reps (range 10–45°)	-
	TRX Push-Up on one Leg	-	3 sets of 6 reps per leg (range 10–45°)
	TRX Standing Hip Drop	3 sets of 10 reps	3 sets of 15 reps
10 min	Exercise with Fitball	in pairs with help	independently
10 min	Stretching	within normal range of motion	within maximum range of motion

Abbreviation: HRmax = maximal heart rate.

## Data Availability

The original contributions presented in this study are included in the article; further inquiries can be directed to the corresponding author.

## Supplementary Materials

The following supporting information can be downloaded at https://www.mdpi.com/article/10.3390/medsci14050538/s1, Table S1: Assumption checks, descriptive statistics, and comparisons for continuous variables.
