# Supplementary material for: A Six-Week Multimodal Functional Training Program Improves Physical Function and Modulates Redox Homeostasis in Older Women
Source: Med Sci (Basel). 2026 Sep 1;14(5):538. doi: 10.3390/medsci14050538 (PMC13609585; doi:10.3390/medsci14050538)
Supplement: Supplementary file 1 [file medsci-14-00538-s001.zip › medsci-4431805-supplementary.pdf]

A Six-Week Multimodal Functional Training Program Improves Physical Function and Modulates Redox Homeostasis in Older Women

Supplementary Table S1. Assumption checks, descriptive statistics, and comparisons for continuous variables

Variable	Normality (Shapiro–Wilk test, *p*)		Before (n = 17), mean [95% confidence interval]	After (n = 17), mean [95% confidence interval]	Before-After differences with 95% confidence interval *	Effect size (absolute value) **	Test used	*p value*
	Before	After						
**Skeletal muscle mass** (kg)	0.226	0.460	22.7 [21.2 – 24.3]	23.0 [21.5 – 24.7]	0.382 [0.246 – 0.519]	d_z = 1.209	paired Student's t-test	0.0006
**Percentage of body muscle mass** (%)	0.130	0.043	32.0 [30.2 – 33.7]	32.5 [30.8 – 34.3]	0.710 [0.447 – 0.938]	r = 0.735	Wilcoxon test	0.0011
**Skeletal muscle mass index** (kg/m^2)	0.292	0.359	6.4 [6.1 - 6.7]	6.5 [6.13 – 6.82]	0.118 [0.065 – 0.170]	d_z =0.617	paired Student's t-test	0.0216
**Body fat mass** (kg)	0.068	0.062	29.2 [23.1 – 35.2]	28.5 [22.6 – 34.4]	-0.959 [0.635 – 1.283]	d_z =0.718	paired Student's t-test	0.0092
**Percentage of body fat mass** (%)	0.498	0.618	39.0 [35.0 – 43.0]	38.2 [34.2 – 42.3]	-1.088 [0.696 – 1.481]	d_z =0.638	paired Student's t-test	0.0181
**Right handgrip strength** (kg)	0.154	0.471	21.7 [20.7 – 22.5]	22.1 [21.2 – 22.9]	0.612 [0.411 – 0.813]	d_z =0.677	paired Student's t-test	0.0131
**Left handgrip strength** (kg)	0.557	0.301	19.2 [17.8 – 20.6]	20.0 [18.8 – 21.3]	0.959 [0.461 – 1.457]	d_z =0.762	paired Student's t-test	0.0063
**Short Physical Performance Battery score**	0.002	0.007	9.1 [8.5- 9.6]	10.0 [9.6 – 10.6]	1.000 [0.567 – 1.904]	r = 0.707	Wilcoxon test	0.0142
**Total antioxidant capacity** (mM Trolox equivalent)	0.713	0.097	6.0 [5.3 – 6.6]	5.8 [5.0 – 6.7]	-0.819 [0.477 – 1.161]	d_z =0.097	paired Student's t-test	0.6935
**Catalase activity** (nmol/min/mL)	0.929	0.385	21.8 [17.8 – 25.7]	26.2 [22.3 – 30.1]	6.551 [3.688 – 9.413]	d_z =0.582	paired Student's t-test	0.0289
**Superoxide dismutase activity** (unit/mL)	<0,0001	<0,0001	2.6 [1.8 – 3.4]	2.5 [1.7 – 3.2]	-0.159 [0.132 – 0.364]	r = 0.394	Wilcoxon test	0.1167
**Glutathione peroxidase activity** (nmol/min/mL)	0.333	0.662	102.9 [98.4 – 107.4]	102.2 [97.4 – 107.0]	-4.943 [2.586 – 7.299]	d_z = 0.101	paired Student's t-test	0.6825
**Total nitrite+nitrate level** (μmol)	0.031	0.0001	17.0 [12.8 – 21.3]	24.2 [12.9 – 35.6]	6.790 [2.216 – 16.990]	r = 0.505	Wilcoxon test	0.0348
**TBARS** (μM malondialdehyde)	0.014	0.499	3.7 [3.3 – 4.2]	4.1 [3.7 – 4.5]	0.750 [0.468 – 1.099]	r = 0.420	Wilcoxon test	0.0934
**Neutrophil granulocytes** (giga/L)	0.169	0.011	4.0 [3.4 – 4.6]	4.0 [3.1 – 4.9]	0.440 [0.291 – 1.306]	r = 0.195	Wilcoxon test	0.4307
**Monocytes** (giga/L)	0.506	0.365	0.5 [0.46 – 0.60]	0.5 [0.44 – 0.59]	-0.076 [0.045 – 0.107]	d_z = 0.101	paired Student's t-test	0.6805
**Oxidative burst capacity** (AUC/1 000 neutrophil)	0.849	0.631	8903 [7476 - 10331]	7933 [6615 - 9252]	-1514 [817.5 – 2210.0]	d_z =0.537	paired Student's t-test	0.0418

*For normally distributed variables, data are presented as mean values with 95% confidence intervals (95% CI), whereas non-normally distributed variables are presented as medians with 95% CIs.
** For normally distributed variables, effect sizes are reported as Cohen's d_z (Cohen's d for paired samples), whereas for non-normally distributed variables, the Wilcoxon effect size (r) is reported.
